# Inspecting teams’ and organisations’ expectations regarding external inspections in health care: a qualitative study

**DOI:** 10.1186/s12913-020-05475-0

**Published:** 2020-07-08

**Authors:** Einar Hovlid, Inger Lise Teig, Kjersti Halvorsen, Jan C. Frich

**Affiliations:** 1 Department of Social Science, Western Norway University of Applied Sciences, P.O. Box 133, 6851 Sogndal, Norway; 2 Department of Global Public Health and Primary Care, University of Bergen, P.O. Box 7800, 5020 Bergen, Norway; 3 Norwegian Board of Health Supervision, Oslo, Norway; 4 Department of Global Public Health and Primary Care, University of Bergen, P.O. Box 7800, 5020 Bergen, Norway; 5 Institute of Health and Society, University of Oslo, P.O. Box 1078, 0316 Oslo, Norway

**Keywords:** External inspection, Organisational change, Quality improvement

## Abstract

**Background:**

There is a gap in the literature regarding what takes place between the announcement of a regulatory intervention, such as an external inspection of a health care organisation, and the inspecting body’s site visit. This study aimed to explore inspecting bodies’ expectations of how inspected organisations should prepare before an external inspection and to elucidate how inspected health care organisations prepare before site visits.

**Methods:**

This qualitative study was based on data from 17 group interviews with a total of 75 participants representing inspection teams, organisation leaders and clinicians in inspected health care organisations. The data were analysed using a qualitative content analysis method.

**Results:**

We identified two approaches to how the inspection teams expected that the inspected organisations should prepare before site visits. In the first approach the inspection teams did not expect any improvement activities to be initiated during this period and focused on identifying inadequacies that the inspected organisations should subsequently improve. In the second approach the inspection teams expected organisations to review their own practices and begin improvement activities if necessary. The inspected organisations responded in different ways to an upcoming site visit, and the organisations’ leaders were important in determining which activities would be initiated. Organisations in which leaders involved clinicians in assessing care delivery tended to initiate action to improve and expected inspection teams to assess their ongoing improvement work and provide guidance on further improvements. Leaders who did not involve clinicians in assessing the quality of care tended to perceive the current quality of care as adequate on the basis of reviewing written guidelines. They did not initiate action to improve care delivery apart from updating written guidelines describing how care should be delivered, and they expected the inspection team to confirm that their current practices were in line with the guidelines and external standards.

**Conclusions:**

To promote anticipatory effects in inspected organisations, inspecting bodies should stress the importance of assessing clinical practice and involving frontline clinical staff and leaders in the assessment and in improvement work before the site visit.

## Background

Initiatives to improve quality and safety in health care may be internal or imposed upon service providers through external requirements and regulations. External governance of health care organisations can serve different purposes, such as improving performance, promoting accountability and providing information about performance to a range of stakeholders [1, 2]. The assessment of an organisation’s performance using externally defined standards is a widely used component in regulatory systems for health care [3]. Governance activities of this type have been described as ‘external inspections’, ‘external reviews’, ‘supervision activities’ and ‘audits’ [1, 4]. This article focuses on external inspection, and we use this phrase to indicate an inspection process based on externally defined standards that is initiated and controlled by an organisation external to the one being inspected [3, 5].

The evidence on the effects of external inspections on quality of care remains unclear, and mechanisms by which external inspections might affect the quality of care are poorly understood [1, 3, 6]. There is a need for more knowledge to increase our understanding of why these effects seem to vary, and to facilitate more effective ways of conducting inspections [1]. Recent research on external inspections has outlined eight theoretical regulatory impact mechanisms, one of which is denoted as ‘anticipatory’ and explained as ‘providers responding to and complying with regulators’ established expectations before any interaction with the regulators takes place’ [7].

Previous research on external inspections has mainly focused on the actual meetings between inspecting bodies and the organisations being inspected, how inspections were perceived by those being inspected and the subsequent consequences for organisational practice. There is a gap in the literature concerning what takes place in the time between the announcement of a regulatory intervention, such as an external inspection of a health care organisation, and the inspecting body’s site visit. A better understanding of the processes that take place before an external inspection may reveal underlying mechanisms for the anticipatory effects of external inspections.

In Norway, the overall responsibility for conducting external inspections of health care services is delegated to the Norwegian Board of Health Supervision (NBHS), a national institution organised under the Ministry of Health and Care Services. The NBHS prioritises thematic areas for inspection based on information about risk and vulnerability. Inspections are then carried out by the 11 county governors in Norway on behalf of the NBHS. Each county governor is responsible for regulatory activities for all providers of health services in his or her geographical region. These regulatory activities can include inspections, handling complaints from patients regarding deficiencies in care delivery, following up on serious adverse events, and providing supervision and guidance. Thus, the county governors have longstanding and constantly evolving relationships with all health service providers in their areas.

The overall purpose of statutory inspections of health care services is to contribute to improving the quality of health services by ensuring that they are provided in accordance with legislative requirements. The standards used for inspections are grounded in legislation and based on two main pillars: Health care services should be safe and effective and provided in accordance with sound professional standards, and all organisations that provide health care services are required to have an internal governance system to ensure that health care services are provided in accordance with the requirements in the legislation.

The inspections are carried out as system revisions based on the International Organization for Standardization’s procedures for system revisions [8]. These procedures have been adapted to the Norwegian context [9]. The inspections consist of four main phases: the development of standards, the announcement of the inspection and collection of relevant documentation and data, the site visit, and reporting and follow-up. For each type of inspection addressing a particular theme, the NBHS develops a detailed written guideline describing how the inspection should be conducted. These guidelines include information about what kind of data should be collected prior to and during the site visit, as well as whom in the inspected organisation should be interviewed. Moreover, the standards used in the inspections are operationalised in relevant audit criteria. These criteria are specific (e.g. ‘The provider needs to screen and identify patients who are at risk of being malnourished’). The guidelines also include guidance on how the inspection teams should assess and judge the different audit criteria. Each inspection is conducted a team consisting of two to four inspectors. Each team is headed by a senior inspector, who has extensive training and experience in performing inspections. The teams are made up of members with clinical knowledge of the area being inspected.

County governors announce an inspection about 8 weeks prior to the site visit by means of a standardised letter, which includes a list of documentation requested from the inspected organisation (e.g. relevant written guidelines and procedures or information about the organisational structure, including a description of the distribution of authority and responsibility). Using this information, the inspection team develops an agenda for the onsite visit. During the visit, the inspection team gathers relevant data, which include interviews with leaders and frontline clinical staff, data from the quality management system (e.g. quality indicators, complaints from patients and reports from internal audits) and relevant data from patient records. The inspection team uses all the data to review the inspected organisation’s performance in relation to the predefined audit criteria. After the site visit, the inspection team writes a report describing the inspected organisation’s performance in relation to the audit criteria. Although the audit criteria are specific, the assessment of whether or not the inspected organisation complies with the standards is to some extent a discretionary judgement. If the inspection team concludes that the performance does not comply with one or more of the audit criteria, the inspected organisation will be required to make necessary changes to become compliant. In that case, the organisation has to develop an action plan and verify that the necessary changes have been implemented. All inspection reports are publicly available on the NBHS’s website.

### Theoretical framework

To maintain reflexivity in the research process, we present the theories that we used as a frame of reference for our research [10].

A key purpose of external inspections is to contribute to improving the quality of care [1]. We understand quality of care as a property of the health systems that deliver care [11]. Accordingly, improving the quality of care depends on changing the performance of the health system; this, in turn, implies organisational change, understood as *any modification in organisational composition, structure or behaviour* [12]. Organisational change is a complex social process that involves a range of different organisational activities [13]. If external inspection has the ability to contribute to improving the quality of care, it should impact the activities involved in organisational change.

Organisational readiness for change, which can be defined as *the extent to which organisational members are psychologically and behaviourally prepared to implement organisational change* [12], is considered a critical precursor to an organisation’s ability to successfully implement change [12, 14, 15]. A basic precondition for creating organisational readiness for change is a realisation that there is something that needs to be changed [14]—a ‘significant difference between the current state or practice and a more desirable state’ [15]. One rationale for using external inspections is to identify gaps between an organisation’s current performance and the expected performance based on the inspection standards [1].

Change is dependent on the commitment to actually address a performance gap [16, 17]. According to Weiner [18], the members of the organisation must collectively value change enough to commit to its implementation and believe that it is urgently needed. It is therefore relevant to explore whether inspections can contribute to creating acceptance of and commitment to change.

The organisational leadership is recognised as an important factor for initiating and facilitating organisational change and improvement [14]. Leadership can influence successful improvements in health care, and the absence of leadership is related to poor quality [19]. Leadership development programmes for physicians have been shown to be associated with improved quality outcomes on a system level [20]. There is a sound basis for claiming that leadership is an important factor in facilitating organisational change [19]. Accordingly, it is necessary to explore to what extent external inspection can contribute to facilitating leader engagement and support for change [1].

### Aim of the study

This study aimed to explore inspecting bodies’ expectations regarding how inspected organisations should prepare before an external inspection and to elucidate how inspected health care organisations prepare before site visits.

## Methods

### Design

We used an exploratory case study design that included six planned inspections and collected data through 17 qualitative group interviews with a total of 75 informants.

### Participants

We used a combination of sampling strategies as described by Miles and Huberman [21]. One scheduled inspection represented one case. We used purposive sampling by including inspections from both primary care and specialised care. Moreover, we also used a conveniences sample as we had to recruit participant from inspections that were already planned by the County Governors.

The NBHS provided us with a list of scheduled inspections. We approached different county governors and the organisations they were scheduled to inspect and asked if they were willing to participate in the research project. We gathered information about the organisation, management and staff composition of the inspected organisations, and we invited strategic and operational leaders as well as representatives from all the involved professional groups to participate. The invitation to participate was made via mail and telephone. Participation was based on voluntary consent. We did not receive any information about the individuals who declined to participate.

In total, we included six cases: three from primary health care settings and three from specialised settings. The selected cases involved inspection teams overseen by five different county governors. The inspected services were nutrition for patients in nursing homes, nutrition for patients receiving home care services, the compulsory treatment of somatic disorders in patients with cognitive deficiencies, stroke treatment and two cases of suicide risk assessment in specialised psychiatric care.

### Data collection

We wanted to explore the organisational processes that take place prior to site visits. Such processes are dependent on interactions among individuals and groups, both in the inspected organisations and in the inspection teams [22]. Group interviews enable interaction between group members during the data collection process, thus resembling the processes we wanted to study. Separate group interviews were conducted for inspection teams and for leaders and frontline clinical staff in the inspected organisations. The characteristics of the included cases are presented in Table 1. The interviews were semi-structured and based on a guide (supplement file). We developed this guide on the basis of previous research on external inspections [2, 23, 24] and our theoretical framework. The interviews lasted approximately 60–75 min and were conducted at the informants’ workplaces. EH (male) and KH (female) conducted the interviews together. EH moderated the discussions, and KH observed, took notes and asked additional questions to elaborate and clarify the group participants’ statements. Except for the two cases of suicide risk assessment in specialised psychiatric care (Cases 5 and 6; Table 1), we conducted three group interviews for each case. For Cases 5 and 6, the inspection team was the same; we therefore conducted one group interview with this team, covering topics relevant to both Case 5 and Case 6, along with separate group interviews with leaders and frontline clinical staff for each case.

**Table 1:** Characteristics of the included cases

Case number	Setting	Inspection theme	Group (N = number of participants in each group interview)	Participants (F = female, M = male)
1	Primary care	Nutrition for patients in nursing homes	Group 1: inspection team (*N* = 2)	Head of inspection team (F) Nurse (F)
			Group 2: leaders (*N* = 6)	Head of municipal health affairs (M) Head of department being inspected (F) Head of subsection (M) Head of subsection (F) Head of subsection (F) Head physician (F)
			Group 3: clinical staff (*N* = 6)	Nurse (F) Nurse (F) Auxiliary nurse (F) Auxiliary nurse (F) Auxiliary nurse (F) Cook (F)
2	Primary care	Nutrition for patients receiving home care services	Group 4: inspection team (*N* = 3)	Head of inspection team (F) Legal advisor (F) Physician (F)
			Group 5: leaders (*N* = 4)	Head of department being inspected (F) Head of subsection (F) Head of subsection (F) Head of subsection (F)
			Group 6: clinical staff (*N* = 6)	Nurse (F) Nurse (F) Nurse (F) Auxiliary nurse (F) Auxiliary nurse (F) Auxiliary nurse (F)
3	Primary care	Compulsory treatment of somatic disorders in patients with cognitive deficiencies	Group 7: inspection team (*N* = 3)	Head of inspection team (F) Legal advisor (F) Physician (F)
			Group 8: leaders (*N* = 4)	Municipality leader for health affairs (M) Head of department being inspected (F) Head of subsection (F) Head of subsection (F)
			Group 9: clinical staff (*N* = 5)	Nurse (F) Auxiliary nurse (F) Auxiliary nurse (F) Auxiliary nurse (F) Physician (M)
4	Hospital	Stroke treatment	Group 10: inspection team (*N* = 2)	Head of inspection team (F) Physician (F)
			Group 11: leaders (*N* = 8)	Head of medical division in hospital (F) Quality advisor for division (F) Head of department being inspected (F) Head of subsection (F) Head of subsection (M) Head of subsection (M) Head physician (F) Quality advisor for subsection (F)
			Group 12: clinical staff (*N* = 6)	Nurse (F) Nurse (F) Nurse (F) Occupational therapist (F) Physician (M) Physiotherapist (F)
5	Hospital	Assessment of suicide risk in specialised psychiatric care	Group 13: inspection team (*N* = 4)	Head of inspection team (F) Legal advisor (F) Nurse (F) Nurse (F)
			Group 14: leaders (*N* = 6)	Head of department being inspected (M) Head of subsection (F) Head of subsection (F) Head of subsection (F) Head of subsection (F) Head of subsection (M)
			Group 15: clinical staff (*N* = 3)	Nurse (F) Nurse (F) Physician (M)
6	Hospital	Assessment of suicide risk in specialised psychiatric care	Group 13: inspection team (*N* = 4)	
			Group 16: leaders (*N* = 2)	Head of department being inspected (M) Head of subsection (F)
			Group 17: clinical staff (*N* = 5)	Nurse (F) Nurse (M) Physiotherapist (F) Physician (M) Psychologist (M)

### Analysis

All interviews were recorded and transcribed verbatim. The transcripts were not returned to the participants for comment. We conducted a thematic content analysis and used a combination of direct and indirect approaches, as described by Hsieh and Shannon [25]. Our initial coding scheme was guided by our theoretical framework. In line with this framework we coded whether the inspections affected organisational composition, structure or behaviour. Moreover we coded whether the inspections contributed to create readiness and commitment to change, and how the leaders responded to the announced inspection. Rather than theory testing, we applied a theory-inspired analysis where the participants’ statements and reflections were in focus. During the analysis, we added codes that emerged during the analysis. All researchers read and discussed the transcripts and agreed upon final categories and their content.

First, we analysed and coded each interview separately to identify higher-order themes and systematically reorganised units of meaning according to these themes for each interview. Second, we condensed and summarised the contents of these nexus of meanings across the interviews within each case. Third, we looked for similarities, differences and patterns across the different cases: between inspection teams, leaders and frontline clinical staff and between primary and specialised care. Using an iterative process of coding, reflecting on the codes and then condensing them, we identified common patterns characterising expectations of and preparations for the inspections [26]. Throughout the analysis, the researchers discussed and compared the thematic content. All participating researchers have extensive training and experience in conducting qualitative research.

### Ethical considerations

The study protocol was approved by the Norwegian Centre for Research Data, which reviewed ethical aspects of the study related to collecting and handling the data (voluntary participation based on informed consent, information provided to participants, the anonymity of informants and the presence of appropriate data storage protocols; project number 39234). All interviewees received written information describing the research project before they gave informed written consent to participate.

## Results

We conducted 17 group interviews with a total of 75 informants (30 leaders, 31 clinicians and 14 inspection team members). Table 1 presents the characteristics of the included cases.

In line with our study aims we identified two types of expectations with corresponding preparatory activities among the inspection teams. Among the inspected organisations, we identified three types of responses and expectations of the inspection teams, and we describe them as three separate themes. In addition to describing these themes, we also analyse the relationship between the three themes and the two types of expectations of the inspection teams.

### Inspection teams’ expectations and preparations

All of the inspection teams stressed the dual purpose of the inspections: 1) to assure that the services were organised and delivered in line with the requirements and 2) to improve the quality of the services provided. However, the inspection teams varied in the emphasis they put on these two aims, and their views were reflected in how they expected organisations to prepare before the site visit. These views were conveyed to the inspected organisations through oral communication prior to the inspection rather than through the standardised letter used to announce the inspections, which focused on practical implications.

Two inspection teams focused primarily on control and quality assurance. The members of these teams emphasised a controlling purpose, seeking to confirm that practices in the inspected organisations followed rules and regulations.

The purpose is to get an overview of how they work in [organisation] with this specific subject. … what doesn’t work according to the requirements and why. In my view, this is the main purpose of the inspection. (Inspection team member).

These two inspection teams did not expect the organisations to improve their routines or services prior to the site visit.

I do not expect anything to happen then [after the notification of inspection] because the measures being launched normally don’t last long. (Inspection team member).

Members of these teams expected leaders in the inspected organisations to take care of practical issues and to inform other organisational staff members about the practical implications of the upcoming inspection.

[We expect] them to facilitate the conducting of the announced inspection and to inform the staff about the subject of the inspection. (Inspection team member).

The inspection teams that oriented towards control initiated activities addressing the control function when preparing themselves for the site visit. They reviewed the documentation sent by the organisations and assessed whether it was in line with the requirements.

I assess the documentation to verify that it is in accordance with the revision criteria. Does the organisation have this written guideline in place? Yes, no, partly. (Inspection team member).

The other three teams had a more proactive approach, expecting the organisations to review their own practices and instigate measures to improve routines and existing practices, if necessary, prior to the site visit. These inspection teams focused on the learning and empowerment of the organisation; for them, control was not the central purpose of the inspection.

After we announce the inspection, I expect the organisation to review their own practice and performance. Yes, I really do—based on our comments. However, measures might be taken or not, but I expect that they at least do a review of their practice. (Inspection team member).

Another inspector made the following comment:

When we announce the inspection, the organisation starts preparing and improving before the site visit. The announcement can be a push for them to start necessary improvement work. (Inspection team member).

The inspection teams that were generally oriented towards improvement also reviewed documentation from the organisations, but the members of these teams were more concerned with establishing an understanding of improvement as a shared aim through their dialogue with the organisations being inspected.

It is really important that the frontline clinical staff understand that we have a shared aim, namely the quality of the services delivered to the patients. … An important part of the inspection is to establish a dialogue with the service provider so that the inspection can contribute to learning and not a feeling of us simply telling them how the service ought to be. (Inspection team member).

### Inspected organisations’ preparations and expectations

A common finding across all the inspected organisations was that, when the inspection was announced, the leaders initiated an assessment of their performance in the area of the inspection. The thoroughness of this assessment and the degree of involvement of the rest of the organisation varied. The leader’s perception of the quality of care in the inspected area determined how the organisation prepared before the site visit. We identified three typical themes describing how organisations responded after being notified about an upcoming inspection visit (Table 2).

**Table 2:** Three themes of responses to an upcoming inspection

1) Leaders and frontline clinical staff perceived the quality of care in the inspected area to be adequate, and, accordingly, no measures were initiated to improve care.	
2) Leaders and frontline clinical staff perceived the quality of care in the inspected area to be inadequate and therefore initiated measures to improve care.	
3) Leaders did not involve frontline clinical staff in assessing the quality of care in the inspected area and perceived the quality to be adequate on the basis of a review of written guidelines. Frontline clinical staff perceived the care to be inadequate and in need of improvement. No measures were initiated to improve care.	

#### Theme one: quality is perceived by leaders and frontline clinical staff as adequate

In the first type of response, leaders and frontline clinical staff perceived the quality of care in the inspected area to be adequate, and, accordingly, no measures were initiated to improve care. The theme of the announced inspection coincided with improvement work that was already underway and that involved the entire inspected organisation. One leader stressed that the announced inspection, in itself, had no impact on their organisational improvement work:

Yes, so one can say that the upcoming inspection is coming as part of a process we have already started. We have worked continuously on this for the last two to three years. (Leader)

The organisation was confident that they were providing adequate quality of care that was in line with the requirements. There was a unanimous perception that the quality of services delivered was good and sound among all types of informants. Because the quality of care was perceived to be adequate, no special measures to improve care prior to the site visit were initiated. This organisation’s expectation of the site visit was primarily that it should confirm the good work they were already doing. One leader characterised the importance of external confirmation of an organisation’s work as follows:

We should not downplay the effect of confirming that an organisation has done something positive because this increases the motivation to do even more. (Leader)

#### Theme two: quality is perceived by leaders and frontline clinical staff as inadequate

In the second type of response, leaders involved the whole organisation in assessing the quality of care in the inspected area. Together, they determined that the quality of care in the area of the forthcoming inspection was inadequate, and measures were initiated to improve this care. A leader described how the whole organisation was involved in this improvement work:

Yes, of course, employees must be informed about this. They are the ones who are out there. They have to know [ … ] It is one thing to facilitate the implementation [of measures]; it is another to run the service and to recognise the goals and the means of how to do this in daily practice. (Leader)

When the internal assessment revealed that the quality of care was not in line with the requirements, both leaders and frontline clinical staff agreed that change was necessary. They viewed the upcoming inspection as an opportunity to improve their practices in advance of the inspection.

[The announced inspection] triggered the focus that we [ … ] had to assess our performance. And when you start looking at it, it becomes interesting; you become more curious. And questions arise [ … ] In relation to this, we have to be honest that we didn’t have control regarding [our internal routines] when the inspection was announced. (Leader)

These organisations started improving their performance and expected that the inspection team would review their current performance, confirm that the organisation was on the right track when it came to improvement work and provide guidance on further improvements during the forthcoming site visit. The guidance role of the inspectors was highlighted as important:

So I think it’s very important that they come and can go through things systematically and give us good advice and guidance on further work. (Frontline clinical staff)

#### Theme three: quality is assessed as adequate solely by leaders

In the third type of response, leaders did not involve frontline clinical staff in assessing the quality of care, relying instead on their own assessment mainly based on reviewing written material and guidelines. One leader described how she prepared for the upcoming inspection:

After having been informed about the inspection, we find all the documents we have. (Leader)

These leaders expected that the inspection would confirm that the quality of care was in line with the requirements.

Surely, we hope to be acknowledged for the good work we think we are doing. (Leader)

The frontline clinical staff, who were not involved in preparing for the inspection, held a different view from that of their organisations’ leaders regarding the quality of care in the area under inspection. A clinician emphasised how she felt ignored by the management in the process prior to the site visit:

I have a feeling that the leaders think that the quality of care of our services is better than it actually is. It [the inadequate quality of care] has been reported to the management several times. (Frontline clinical staff)

Frontline clinical staff who were not involved in the inspection preparation voiced their frustration in the interviews, pointing to their perception that the management was only concerned with written documentation and not with the actual care delivered to patients. In some cases, tensions between the staff members and the management arose.

Yes, as we see it here, the management is only stressing correct written guidelines. And then I get aggressive because it’s the ordinary work in the unit that matters. (Frontline clinical staff)

These frontline clinical staff members expected that the inspection would reveal that the quality of care was inadequate and thus make the leaders realise this fact. They also expected the inspectors to report the actual state of their practices. In the view of these staff members, if the outcome of the inspection were to fail to address the substandard performance, the inspection would also be seen as inadequate.

But we know that it [the quality] is not optimal, so if the outcome is described as brilliant, I think the inspectors have done an inadequate job. (Frontline clinical staff)

#### Organisations’ expectations of the inspection teams

The inspected organisations had expectations regarding how the inspections should be conducted and especially regarding which competences inspection teams needed to possess to conduct an expedient inspection. In all of the studied cases, the inspected organisation representatives highlighted the need for three types of core competences in the inspection teams: professional knowledge about the particular field being inspected, knowledge about rules and regulation, and knowledge about the organisation and its context.

They [the inspection team] need to know about the rules and regulations that apply. They also need professional health competence and knowledge about the context in which the inspection and service delivery take place. (Leader)

They [the inspection team] need to have health care competence. They should know our field. (Frontline clinical staff)

Primary care and specialised care organisations differed regarding their expectations regarding the forthcoming inspection. Compared with primary care organisations, specialised care organisations were more concerned about the inspection process being transparent and about the validity and reliability of the conclusions.

There needs to be a standardised procedure [for the inspection] that makes it possible to compare findings. The inspection must be based on a thorough and robust method that produce valid and reliable conclusions so that we can compare the findings with other inspections. (Leader)

Primary health care organisations, in contrast, were more concerned about the communicative and relational skills and competences of the inspectors.

[that] they need to be kind, especially with the staff—to meet them with respect (Leader)

I hope that they have social competence—I mean, that one does not become so frightened that one cannot do anything, that they manage to make us display what we actually can. (Leader)

## Relationship between the inspection team’s approach and the organisation’s response

**Fig. 1:**
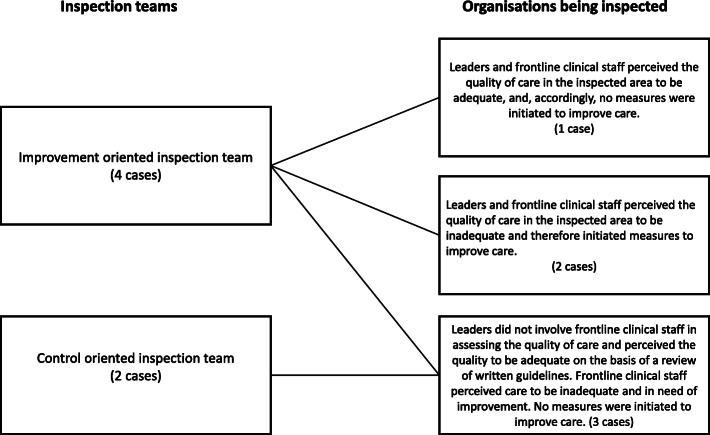
Relationship between the inspection team’s approach and the organisation’s response

Figure 1 shows how the two inspection approaches (control and improvement) relate to how the inspected organisations prepare. In cases where the inspection team put the primary emphasis on a controlling function, few preparations were instigated, whereas more activities directed at improving practice were initiated in cases where the inspection team’s focused was on improvement.

## Discussion

### Main findings

Our data indicate that inspection teams’ expectations and communication with inspected organisations can influence how the organisations prepare before the site visit.

Organisations in which the leaders involved frontline clinical staff in assessing care delivery were able to gain a better understanding of their current practice and consequently of the need to improve. These organisations tended to initiate action to improve and expected the inspection team to assess the organisation’s ongoing improvement work and provide guidance on further improvements. Leaders who did not involve clinicians and instead based their assessments solely on reviewing written guidelines did not initiate action to improve care and expected the inspection team to confirm that their current practices were in line with the guidelines and requirements.

A key factor explaining how the inspection teams and the inspected organisations related to one another is how language was used and which concepts the inspectors drew on prior to the inspection. How the inspectors communicated their mission, intentions and selected outcomes to investigate influenced how the organisations prepared for the visit and whether they initiated internal action for improvement. When inspection teams displayed a controlling intention and communicated expectations that were primarily about adherence to the rules and regulations, fewer improvement measures were initiated prior to the site visit. In contrast, when expectations regarding the improvement of current practices were communicated using a language of guidance rather than of control, more involvement and engagement were seen among both leaders and staff members in the inspected organisations.

### Comparison with existing literature

Previous research has shown that involving those who are inspected can be a critical factor for implementing change [7]. Staff involvement in accreditation processes has been shown to be associated with higher quality results [27]. When an inspection involves only part of the organisation, there is a risk that those who are not involved will not buy into the organisation’s potential for change [28]. We found that the degree of staff involvement in preparing for inspections varied, and few changes were made in clinical care when clinical staff involvement was low.

Leaders can play an important role when it comes to initiating general improvement activities in health organisations [19]. We found that leaders in the inspected organisations were crucial in determining how their organisations prepared for an announced inspection. This finding is in line with previous research indicating that leader engagement during inspections can provide direction to the improvement process and facilitate the involvement of other staff members [29, 30] and that leader engagement is associated with perceived improvement results [31, 32].

### Implications

Social factors such as the communication and relationships between inspectors and health professionals can affect the impact of inspections [7]. Our findings shed light on how communication and different expectations regarding site visits might affect inspection outcomes.

We identified variation in inspection teams’ emphasis of the control or improvement aspect of the inspections, as well as in inspected organisations’ expectations of the outcome of the inspections. Inspection teams need to keep in mind that inspected organisations may have different preconditions for utilising the inspection findings, depending on their preparations and readiness for change.

Organisational readiness for change is considered a critical precursor for implementing change [12, 15]. We found that the announcement of an inspection could prompt inspected organisations to assess their own practices and to subsequently initiate improvement measures when they identified substandard practices. Hence, by prompting assessments of clinical practices, inspections contributed to organisations’ readiness for change. Inspection teams’ communication with the inspected organisations prior to site visits should therefore emphasise the involvement of clinicians and the assessment of actual clinical practice, rather than only written guidelines, to facilitate readiness for change and the improvement of care.

Organisations that expect confirmation of their current practices without having sufficiently reviewed these practices might not be ready for change because they have not identified performance gaps themselves. If the inspection identifies performance gaps in such a setting, the inspection team should take into account that simply making the organisation aware of these gaps might not be sufficient to create readiness for change. Change is dependent on the commitment to actually address the performance gap [16]. Weiner [18] argued that the members of an organisation must collectively value change enough to commit to its implementation. It is therefore important that inspection teams communicate and convey inspection findings in a way that contributes to creating acceptance and commitment to change.

### Strengths and limitations

The main strength of our study was that we explored expectations and preparations in an inspection cycle before the site visit, in contrast to most previous research, which has examined these topics only in retrospect. Moreover, we collected and compared data from the perspectives of three different groups: inspection teams, organisation leaders and clinicians. We did not have access to performance data, and we therefore do not know how or to what extent the changes that the inspected organisations made actually affected the delivery of care. Our exploratory case study covered upcoming inspections of six different organisations. Although we used purposive sampling to select a varied set of cases, all of the cases were set in a statutory inspection context.

We included cases from different healthcare settings. The requirements that apply for the types of issues that were addressed in the different inspections do also vary. There are for instance more explicit requirements applying to suicide risk assessment compared to nutrition in a home care setting. These contextual differences can contribute to explain the differences in expectations and response that we have observed in our study. More research is needed to explore these matters further and to explore whether our findings are relevant for different inspection contexts.

## Conclusions

The anticipatory effects of inspections seem to be dependent on leader engagement and the involvement of clinicians in assessments of clinical practice. Inspecting bodies may have differing expectations regarding what inspected organisations should do ahead of a planned site visit. To promote anticipatory effects in inspected organisations, inspecting bodies should stress the importance of assessing clinical practice and involving frontline clinical staff in the assessment of current practice and in improvement work before the site visit. Moreover, it is also important that inspection teams communicate with inspected organisations in a way that contributes to creating acceptance and commitment to change.

## Supplementary information

**Additional file 1.**

## Data Availability

No supplementary data are available.

## Abbreviation

NBHS: Norwegian Board of Health Supervision
