# Supplementary material for: Inspecting teams’ and organisations’ expectations regarding external inspections in health care: a qualitative study
Source: BMC Health Serv Res. 2020 Jul 8;20:627. doi: 10.1186/s12913-020-05475-0 (PMC7346447; doi:10.1186/s12913-020-05475-0)
Supplement: Supplementary file 1 — Additional file 1. [file 12913_2020_5475_MOESM1_ESM.docx]

Guide for group interviews

### Inspection team

What is the purpose and aim of the planned inspection?

What is the background for the inspection?

How did you choose the topic for the inspection and whom to inspect?

How do you plan the inspection?

How did you communicate with the organization to be inspected?

What kind of information and data do you gather before the site visit?

How do you use this data and information?

What do you expect the organizations to do when you announce the inspection?

To what extent do the organization you inspect have prior knowledge and experience with inspections?

How can you as an inspection team contribute towards improving patient safety and quality of care during the inspection process?

What are the drivers in the inspected organizations for complying with the requirements?

Which factors do you consider important for conducting an inspection that can be of value for the organization being inspected?

Is there anything that can go wrong during the inspection?

How do you handle potential anxiety or nervousness amongst personnel in the inspected organization?

Anything that you perceive as important that we have not addressed so far?

### Leaders and frontline clinical staff in organization being inspected

What is the purpose and aim of the planned inspection?

Why do you think that your organization was chosen to be inspected?

How do you perceive the standard of your performance in the area that is going to be inspected?

Have you yourself identified any specific quality problems in this area and have you done anything to improve them?

Do you think that the inspection can contribute to improve the quality of services, and if so how?

What is on stake when you know that there will be an inspection?

What information and data did the inspection team ask you to provide?

How did you gather this information, and was it readily available?

Is there anything that can go wrong during the inspection?

Are you familiar with the legal requirements of the area being inspected, and how do you perceive these requirements?

Are your performance in line with the requirements?

Do you think that you need to implement changes following the inspection?

What have you done after you received the letter announcing the inspection?

What do you consider to be important leader task during the inspection process?

Can you describe a successful inspection process from your point of view?

Which factors are important for a successful inspection?

Anything that you perceive as important that we have not addressed so far?
